# The Effect of Non-Measured Points on the Accuracy of the Surface Topography Assessment of Elements 3D Printed Using Selected Additive Technologies

**DOI:** 10.3390/ma16010460

**Published:** 2023-01-03

**Authors:** Paweł Zmarzły, Tomasz Kozior, Damian Gogolewski

**Affiliations:** Faculty of Mechatronics and Mechanical Engineering, Kielce University of Technology, al. Tysiąclecia Państwa Polskiego 7, 25-314 Kielce, Poland

**Keywords:** non-measured points, surface topography, 3D printers, additive technology, optical measurement

## Abstract

The paper presents the results of research aimed at evaluating the surface topography including the analysis of the number of unmeasured points of the samples 3D printed using four additive technologies (i.e., PolyJet Matrix, fused deposition modeling, selective laser sintering, and selective laser melting). The samples were made in three variants of location on the printing platform of 3D printers. Measurements of the samples’ surface topography were carried out using a Talysurf CCI Lite optical profilometer and a Talysurf PGI 1230 contact profilometer. The percentage of non-measured points for each sample and the parameters of the surface topography were determined. Then, the non-measured points were complemented and the topography parameters for the corrected surface were recalculated. In addition, to perform comparative measurements, each surface was measured using a contact profilometer Talysurf PGI 1230. Preliminary results of the research showed that the measurement of the surface topography of the samples made using selective laser sintering technology with the Taysurf CCI optical measuring system is very unreliable, as the number of non-measured points for the analyzed samples was higher than 98%. The highest accuracy of optical measurement was obtained for PJM technology and three variants of location on the printing platform of the 3D printer.

## 1. Introduction

Additive technologies are becoming increasingly popular in the ongoing Industry 4.0 revolution [1]. In the beginning, they were mainly used for the construction of prototypes and models. However, currently, due to the decrease in the prices of 3D printers and printing materials, they are used in various sectors of the economy (i.e., in foundries, automotive, space, textiles, and medicine [2,3,4,5,6,7]. Along with the dynamic development of 3D printers, the dimensional and shape accuracy and mechanical strength of the elements printed using additive technologies are constantly improving [8,9]. This allows for the production of finished (precise) products without the need for further machining [10] as well as using the tools of reverse engineering [11]. Therefore, there is a need for a comprehensive analysis of the surface topography of such elements using contact or optical profilometers [12].

The main materials used in additive manufacturing technologies for the printing of functional elements are plastics, but recently also metal alloys (steel, aluminum alloys) [13,14,15]. In the case of some types of additive technologies and the materials used for 3D printing, there is a problem with the measurement and analysis of the surface topography. Additionally, the use of functional coatings can pose measurement problems [16,17]. For example, the elements made using SLS technology (selective laser sintering) have a low yield point [18]. The surface topography of such elements should not be analyzed using contact methods due to the risk of scratching (destruction) the measured surface [19]. In such cases, the use of non-contact, optical methods of measurement is recommended [20]. However, due to the layered nature of the 3D print and anisotropy of the surface, there is a very large number of non-measured points. This problem technically makes it impossible to use this type of measuring instrument for the analysis of surface stereometry. Therefore, other methods of evaluating the surface topography of elements made using some additive technologies should be sought.

The main purpose of the research presented in this paper was to analyze the number of non-measured points for selected additive technologies by taking into account the 3D printing direction, and to assess the impact of non-measured points on the accuracy of the surface topography parameters.

There have been some research projects dealing with the problem of measuring the surface topography of some materials using optical profilometers, however, these projects have mainly been based on elements made using conventional methods of production [21]. The study in [22] presented research aimed at assessing the impact of undetected points on the accuracy of the measurement of the surface of elements subjected to various surface machining methods (i.e., milling, honing, and polishing). In addition, the effect of the lighting intensity setting of the Talysurf CCI measuring device on the number of undetected points was tested. The research has shown that even a small number of undetected points significantly affects the accuracy of the 3D roughness parameter determination. The authors in [23] analyzed the surface topography of the faces of seal rings made of silicon carbide and carbon graphite by using three different measurement systems. A contact profilometer, an optical profilometer, and an atomic force microscope were used for comparative analysis. The research showed that for the silicon carbide ring, surface texture measurements using atomic force microscopy and optical instruments more accurately represented the actual topography than the measurements determined by the stylus profilometer. A research project was also performed to determine the optimal method for measuring the surface roughness in medical applications (i.e., assessing the surface roughness of a femoral head) [24]. The test results proved significant discrepancies between the values of the 3D roughness parameters (St, Ssk, and Sku) by using optical and contact profilometers. In [25], practical issues related to optical measurements were presented. In [26], they presented the experimental research of a preloaded asymmetric multi-bolted connection.

The analysis of the literature showed that there are many works related to the analysis of contact and optical methods of surface topography measurement. However, there have been no research projects on the selection of the optimal method for evaluating the surface topography of elements 3D printed using commonly used additive technologies. Moreover, most works on optical measurements do not mention the number of non-measured points, which is a critical issue when it comes to the accuracy of determining the surface topography parameters. It should be noted that the so-called “good measurement practice” is not always adhered to and metrologists “blindly” trust the software, which does not indicate the percentage of the measured points. Sometimes, especially in industrial applications, previously prepared measuring programs (templates) are used to control the surface of mass-produced elements. Then, the non-measured points are filled in automatically (without the operator’s involvement). This is an important problem that has been overlooked in many publications.

Therefore, the present paper presents studies aimed at the evaluation of the percentage of non-measured points depending on the type of additive technology, printing material, and the way the samples are located on the 3D printing platform in the XZ axis. In addition, an assessment of the impact of supplementing non-measured measurement points using DigitalSurf’s MountainsMap^®^ software on the values of selected types of surface topography parameters was performed. The types of tested parameters were selected in such a way as to define in detail the nature of the surface irregularities. To obtain reference values for the analyzed surface topography parameters, each surface was tested using a Talysurf PGI 1230 contact profilometer. The contact measurement details were compared with the results of the optical measurements.

## 2. Materials and Methods

The experimental research conducted in relation to this study was carried out in two stages. The first stage involved making samples using four additive technologies (PJM, FDM, SLS, SLM), taking into account three directions of the models’ location on the printing platform in the XZ axis (i.e., Pd = 0°, Pd = 45° and Pd = 90°). The second stage involved surface topography measurements using two measuring systems: a Talysurf CCI optical profilometer and a Talysurf PGI 1230 contact profilometer.

### 2.1. Sample Preparation

**Figure 1:**
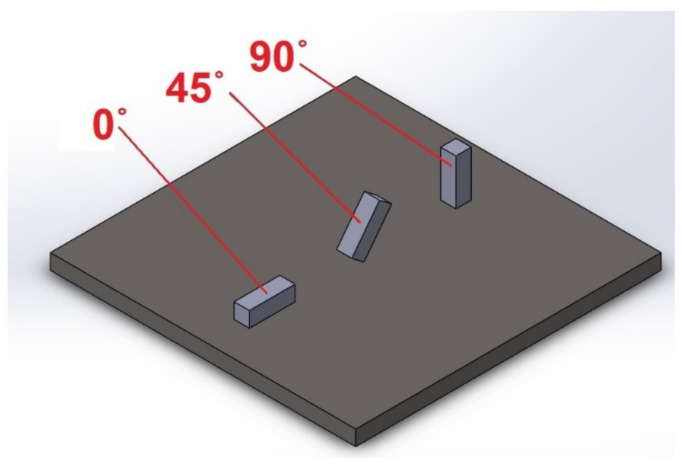
Location of the samples on the 3D printing platform.

The below CAD model was used to make samples for all of the analyzed additive technologies. Then, the CAD model was approximated using a triangle mesh into an STL (stereolithography language) file. The approximation parameters were selected in such a way that the accuracy of the STL model was greater than the accuracy of the most accurate printer used to print the sample (PJM printer). Due to the geometry of the samples, each possible notation approximated the 3D models with 12 triangles. The STL model prepared in this way was sent to each 3D printer. Samples located on the printing platform were made for each printing technology in three variants in the XZ axis (Figure 1).

**Figure 2:**
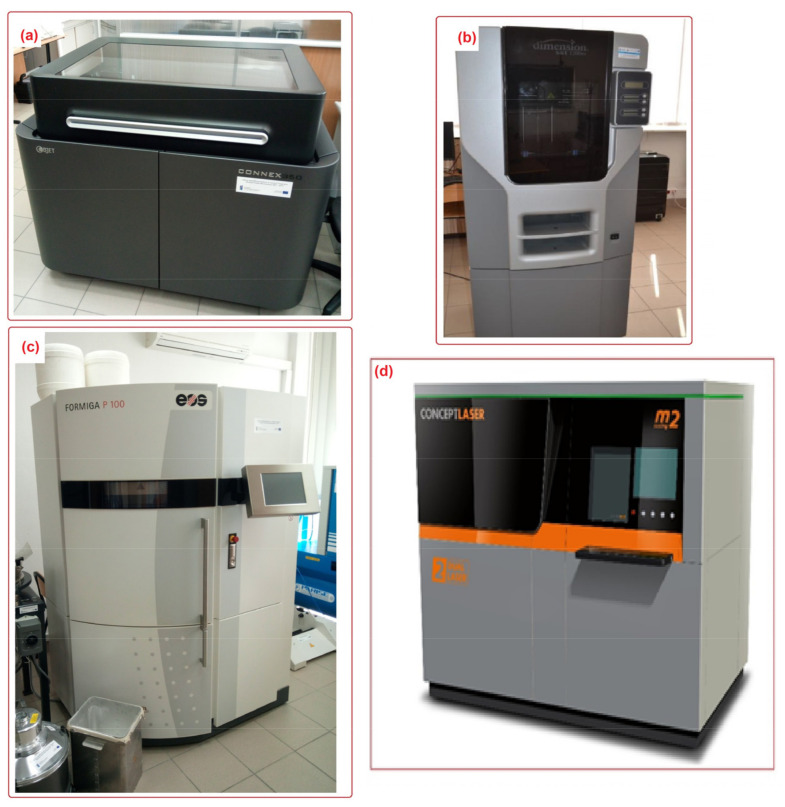
The 3D printers used to 3D print the samples. (**a**) Connex 350, (**b**) 3D Dimension 1200es, (**c**) Formiga P100, (**d**) Concept laser M2.

A different material and a different method of connecting individual layers of the material were used for each of the researched printing technologies. Samples were printed using PolyJet Matrix (PJM) Technology with a Stratasys Connex 350 machine and liquid resin—FullCure 720 material. The layer thickness used was Lt = 0.016 mm. To implement fused deposition modeling (FDM) technology, a 3D printer Dimension 1200es (Stratasys) and ABS P430 material were used. The thickness of a single layer of the printing material was Lt = 0.254 mm. A Formiga P100 printer (0.1 mm layer thickness) was used to print the sample using selective laser sintering (SLS) technology. To make samples with this technology, polyamide powder PA2200 based on polyamide PA12 was used. A Concept laser M2 printer was used to print the sample using powder laser sintering (SLM) technology. The printing material was a 316L tool steel powder. The thickness of a single layer of the material applied using SLM technology was Lt = 0.060 mm. Figure 2 shows the 3D printers used for the research.

Due to the different chemical and physical properties of the materials used to print the samples, the selected mechanical properties of the tested materials are given in Table 1.

### 2.2. Metrological Measurements

The first step of the research procedure was the measurement of the surface topography of the samples using a Talysurf CCI optical profilometer. This measurement system is equipped with a set of lenses (×2.5; ×10; ×20; ×50) and enables the analysis of the surface texture with a vertical resolution of up to 0.01 nm. The horizontal resolution achieved by the device was of the order of 0.33 µm (for a ×50 lens). The measuring range in the Z axis was 2.2 mm. Measurements of samples were made on an area of 1.66 mm × 1.66 mm. The sampling interval was as follows: ΔX = 1.62 µm, ΔY = 1.62 µm. Based on the measured surfaces, the parameters of the 3D surface topography (i.e., Sa, Sq, Sz, Ssk, Sku) were determined. Parameter Sa is known as the arithmetical mean height and it expresses, as an absolute value, the difference in height of each point compared to the arithmetical mean of the surface. Parameter Sq is the root mean square height and it represents the root mean square value of ordinate values within the definition area. The parameter Sz indicates the maximum height. This parameter is defined as the sum of the largest peak height value and the largest pit depth value within the definition area. Ssk (skewness) values represent the degree of bias of the roughness shape (asperity). The kurtosis parameter (Sku) value is a measure of the sharpness of the roughness profile.

The non-measured surface points were then filled using MountainsMap^®^ software options called “smooth shape calculated from the neighbors—optimize for space points” to assess the effect of the procedure of filling the non-measured points on the values of the 3D roughness parameters. The parameters of the surface topography for the same surfaces were determined again once the procedure of filling the points of the non-measured surfaces was complete. In addition, to obtain reference results (reference values), each surface at the same locations was measured using the Talysurf PGI 1230 contact profilometer. Due to the laser interferometer mounted in the “z” axis of the Talysurf PGI 1230 measurement system, the measurement resolution in the z axis was up to 0.8 nm. The measuring range in the “z” axis of Talysurf PGI 1230 was 12.5 mm. The measurement parameters were selected in such a way as to correspond to the measurements carried out using the Talysurf CCI optical system.

## 3. Results and Discussion

Table 2 presents the measurement results obtained using the Talysurf CCI optical device without filling the non-measured points (before filling) and after applying the filling procedure called “smooth shape calculated from the neighbors”. In addition, the table includes the type of additive technology used and the 3D printing direction (Pd).

When analyzing the results presented in Table 2, one can note that some surfaces of the elements printed using selected additive technologies were “immeasurable” using the Talysurf CCI optical profilometer (about 1% of the measured surface). For example, for selective laser sintering (SLS) technology, depending on the selected print direction, the number of non-measured points was in the range of 98.8–99.6%. This is closely related to the nature of the surface of the elements printed using SLS technology and the properties of the material in terms of light absorption. The sintered powder creates a surface that scatters light to a large extent, which makes it much more difficult to carry out optical measurements. This means that the selected surfaces technically cannot be measured using optical devices and other measurement methods (e.g., contact measurements) should be sought. The same is true for the most popular additive technology, namely, FDM technology. Here, for the printing direction Pd = 45°, the number of non-measured points was 89.8%, while for the 3D printing direction of Pd = 90° (i.e., perpendicular to the surface of the printing platform), the number of non-measured points was 60.4%. Considering the results obtained for selective laser melting (SLM) technology, relatively divergent results were obtained depending on the printing direction. The best results were obtained for the printing angle of Pd = 45° (30.1%), and the worst for Pd = 0° (72.9%). Due to the method of laying successive layers of material by the printer, the direction of Pd = 45° is the most recommended. The best results were recorded when using photo-curing of liquid polymer resin technology—PJM. Using this technology, for the print angle Pd = 90°, only 1.5% of the points were not measured. It should be added that the surface of the elements printed using PJM technology is highly reflective, which greatly facilitates the measurements performed with the Talysurf CCI profilometer. Therefore, it can be concluded that optical measurements can be used to analyze the topography of the surface of elements printed using PJM technology.

Considering the impact of the procedure of filling the non-measured points, one can note a clear change in the values of the surface topography parameters depending on the percentage of unmeasured points. For the SLS technology, where the level of undetected points was the highest and fluctuated around 99%, a clear decrease in the surface topography parameters Sa, Sqm, and Sz was observed. This was clearly visible, especially for the print angle Pd = 0° and the parameter Sa, where the reduction was about 27%. Similar trends were noted for FDM technology and the printing direction Pd = 0°, where the number of non-measured points was 88.8%; however, in this technology, the reduction in the surface topography parameters was much lower. This may be due to the fact that clear and regular paths of the printing material are visible when using FDM technology, which resulted in a higher accuracy of the mapping of the non-measured points.

For the other technologies, where the number of undetected points was significantly smaller, the values of parameters Sa, Sq, and Sz increased. It should be noted that for the PolyJet Matrix Technology, where the surfaces were measured with the highest accuracy (the smallest number of measured points), the procedure of filling the non-measured points technically did not affect the values of the surface topography parameters. When analyzing the parameters of surface skewness Ssk and kurtosis Sku, it can be concluded that as a result of the procedure of filling the non-measured points, similar tendencies (trends) were observed as for the parameters of height (i.e., Sa, Sq, and Sz). However, the interpretation of the surface based on the Ssk and Sku parameters, despite a slight change in their values, did not change significantly.

When analyzing the results presented in Table 2 as a whole, it can be concluded that in the case of hard-to-measure surfaces, where the number of non-measured points is high, the procedure of filling them significantly reduces the values of the surface topography parameters. If this fact is not taken into account when analyzing the surface topography of 3D printed elements, erroneous conclusions concerning the nature of the surface can be drawn.

However, referring to the results of the surface topography measurements using the Talysurf PGI 1230 contact profilometer (see: Table 3), one can conclude that the highest values of Sa and Sq roughness parameters were obtained for FDM technology and the printing direction of Pd = 45°. These results are inconsistent with the results of the roughness measurement using the Talysurf CCI profilometer, where the highest values of the Sa and Sq parameters were measured when SLS technology was used. The lowest values of the Sa and Sq parameters were recorded for the technology of PolyJet Matrix (PJM). This is consistent with the results obtained when using the optical profilometer. It is important to say that for this technology, the highest number of points was detected. This was confirmed by the fact that the number of non-detected points directly affects the quality of the surface topography assessment, and consequently, the values of the roughness parameters. When analyzing the parameter determining the maximum height of the surface topography (maximum height), it should be stated that for both the optical and contact measurements, the maximum value of the Sz parameter was obtained for samples printed using SLS technology at the sample position of Pd = 0°.

It should be added that for all of the analyzed additive technologies, the smallest values of the amplitude parameters (Sa and Sq) were obtained for the printing angle of Pd = 90°. This is closely related to the way the successive layers of the printing material are formed. Therefore, this way of positioning the models on the printing platform is recommended to lower the roughness of the surface. A certain disadvantage of such a location of the element on the printing platform is the long printing time.

**Figure 3:**
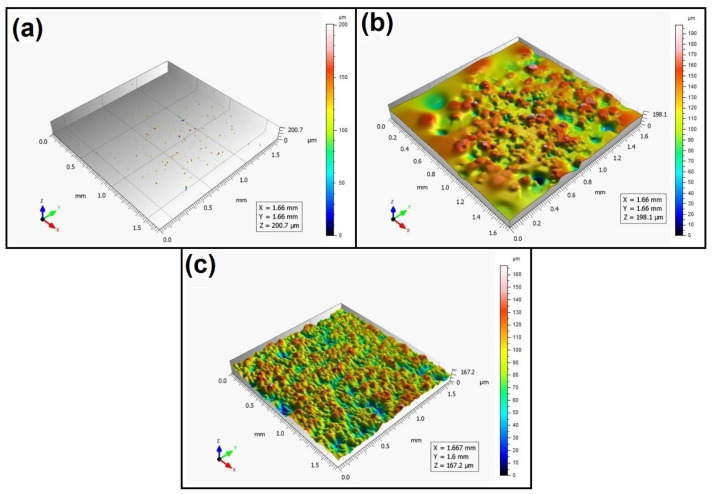
Surface topography of the element 3D printed using SLS technology (Pd = 0°). (**a**) Talysurf CCI—without after filling non-measured points, (**b**) Talysurf CCI—after filling non-measured points, (**c**) Talysurf PGI 1230.

**Figure 4:**
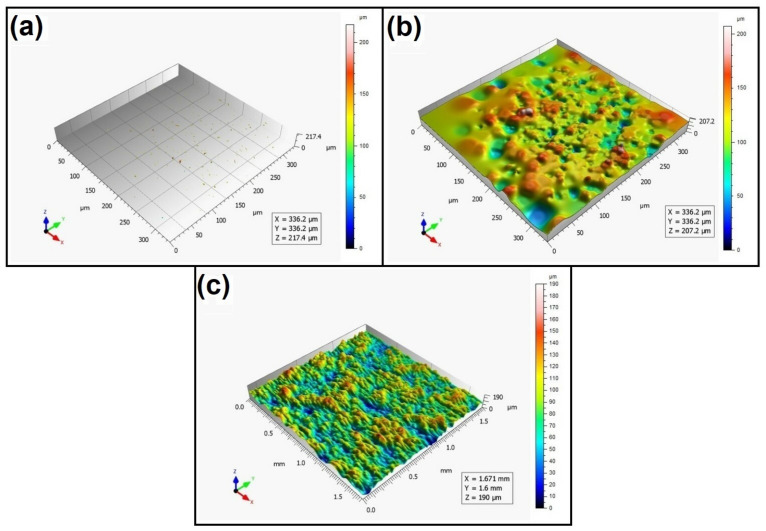
Surface topography of the element 3D printed using SLS technology (Pd = 45°). (**a**) Talysurf CCI—without filling non-measured points, (**b**) Talysurf CCI—after filling non-measured points, (**c**) Talysurf PGI 1230.

**Figure 5:**
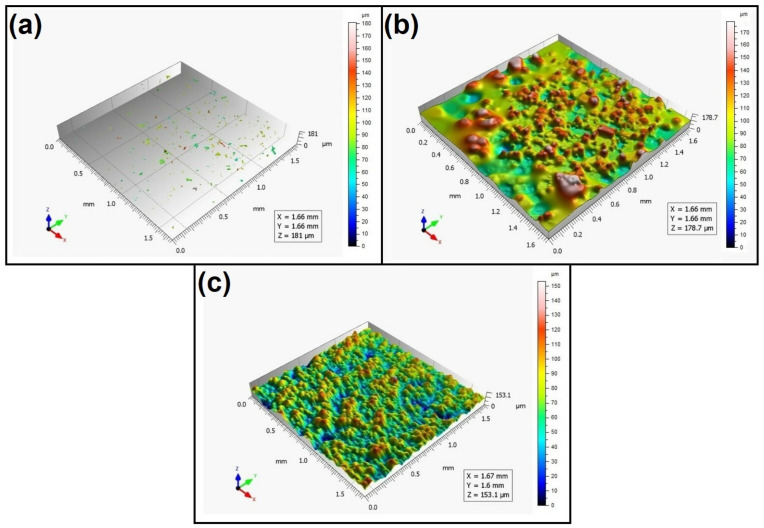
Surface topography of the element 3D printed using SLS technology (Pd = 90°).(**a**) Talysurf CCI—without filling non-measured points, (**b**) Talysurf CCI—after filling non-measured points, (**c**) Talysurf PGI 1230.

**Figure 6:**
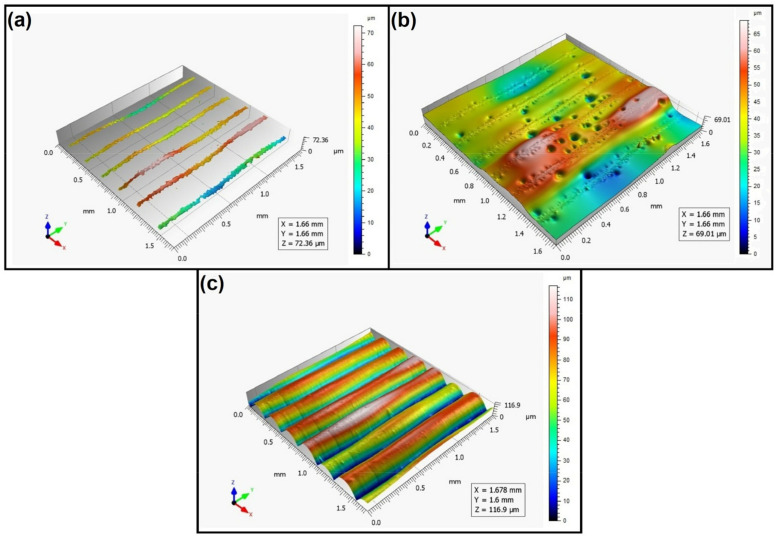
Surface topography of the element 3D printed using FDM technology (Pd = 0°). (**a**) Talysurf CCI—without filling non-measured points, (**b**) Talysurf CCI—after filling non-measured points, (**c**) Talysurf PGI 1230.

**Figure 7:**
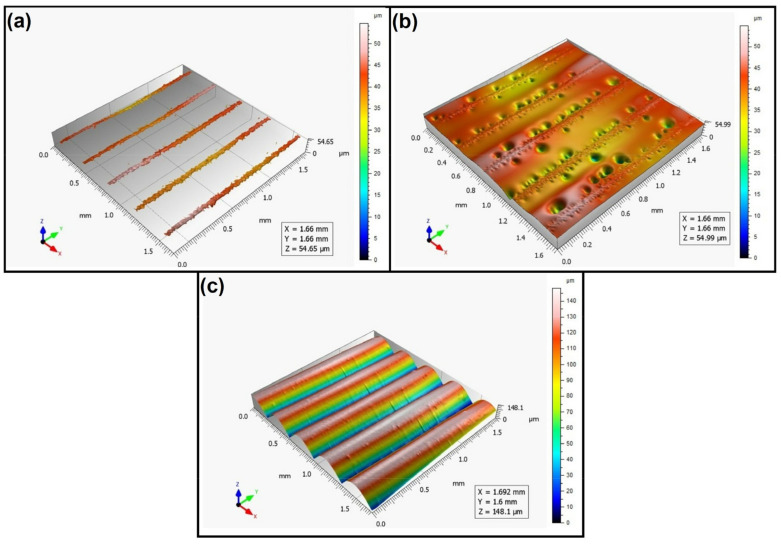
Surface topography of the element 3D printed using FDM technology (Pd = 45°). (**a**) Talysurf CCI—without filling non-measured points, (**b**) Talysurf CCI—after filling non-measured points, (**c**) Talysurf PGI 1230.

**Figure 8:**
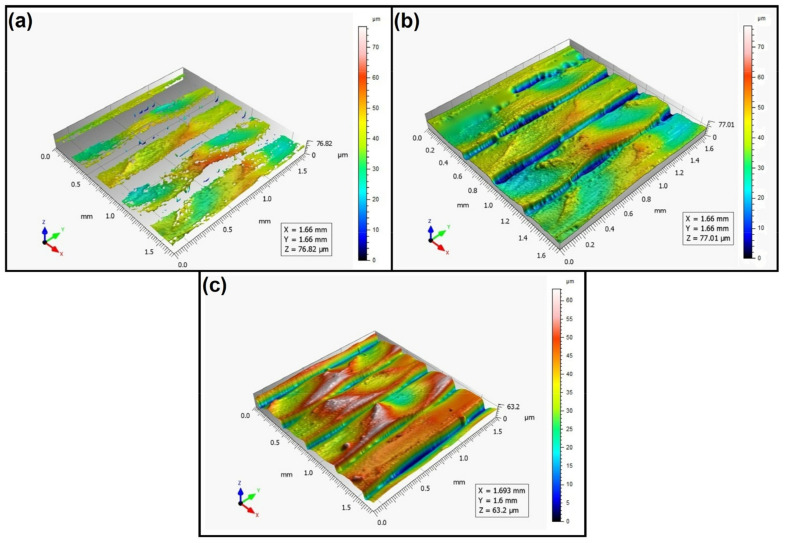
Surface topography of the element 3D printed using FDM technology (Pd = 90°). (**a**) Talysurf CCI—without filling non-measured points, (**b**) Talysurf CCI—after filling non-measured points, (**c**) Talysurf PGI 1230.

When analyzing the values of the skewness, namely, the Ssk parameter measured by the contact method, it can be concluded that in SLS technology and the print directions Pd = 45° and Pd = 90°, the values of the Ssk parameter were close to zero. This means that the considered surfaces are symmetrical (i.e., there is a large number of individual peaks and valleys). This can be proven by the surface topography views given in Figure 3, Figure 4 and Figure 5. A similar effect was visible when using PJM Technology for the Pd = 90° printing directions. In the case of FDM technology, the values of the Ssk parameter were negative for all the analyzed print directions. This indicates that during printing, plateau-shaped surfaces were created (i.e., with single depressions and a concentration of material around the peaks, see Figure 6, Figure 7 and Figure 8). A similar concentration of material around individual peaks was noted for SLS technology and the printing direction of Pd = 0°. However, when considering the surface of the elements printed of metal powder using SLM technology for the printing directions Pd = 0° and Pd = 45°, it can be concluded that the surfaces had few peaks and the 3D printing material was concentrated around the valleys. This was also confirmed by the kurtosis parameter interpretation (i.e., Sku). For SLM technology and the printing directions Pd = 0° and Pd = 45°, the Sku parameter values significantly exceeded 3. As a result, the surface was full of sharp peaks.

**Figure 9:**
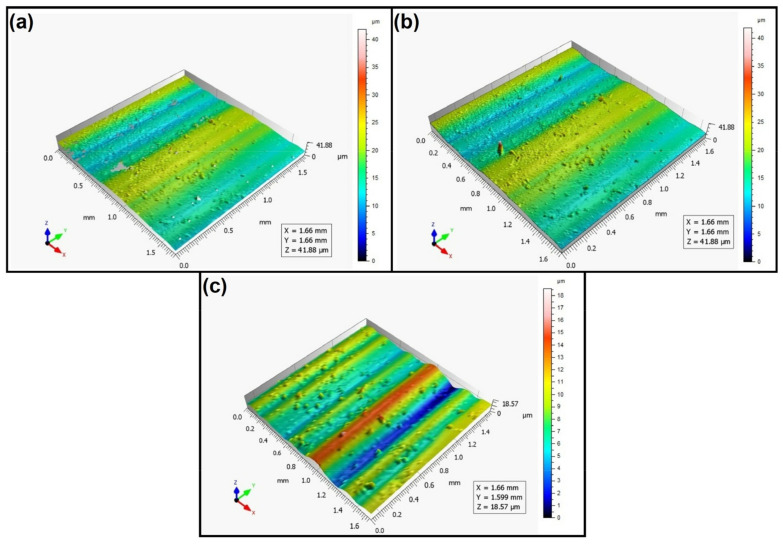
Surface topography of the element 3D printed using PJM technology (Pd = 0°). (**a**) Talysurf CCI—without filling non-measured points, (**b**) Talysurf CCI—after filling non-measured points, (**c**) Talysurf PGI 1230.

**Figure 10:**
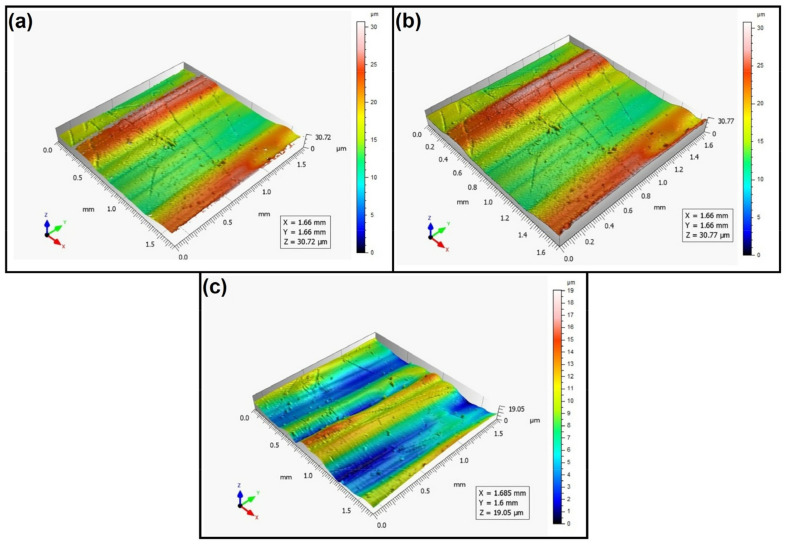
Surface topography of the element 3D printed using PJM technology (Pd = 45°). (**a**) Talysurf CCI—without filling non-measured points, (**b**) Talysurf CCI—after filling non-measured points, (**c**) Talysurf PGI 1230.

**Figure 11:**
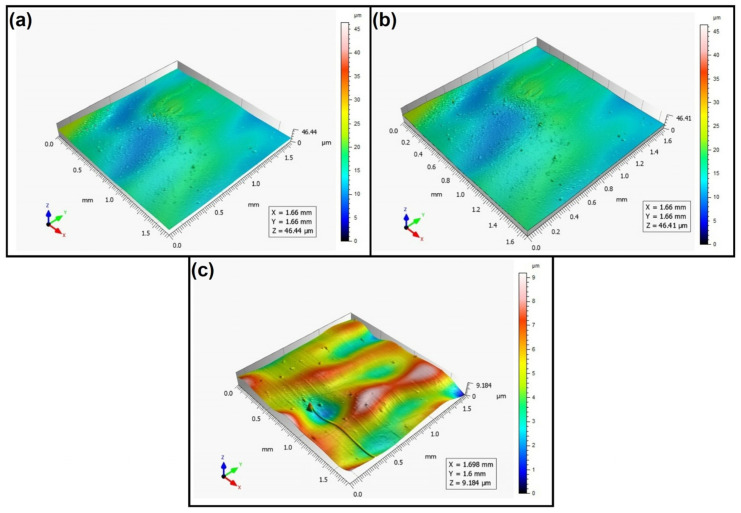
Surface topography of the element 3D printed using PJM technology (Pd = 90°). (**a**) Talysurf CCI—without filling non-measured points, (**b**) Talysurf CCI—after filling non-measured points, (**c**) Talysurf PGI 1230.

**Figure 12:**
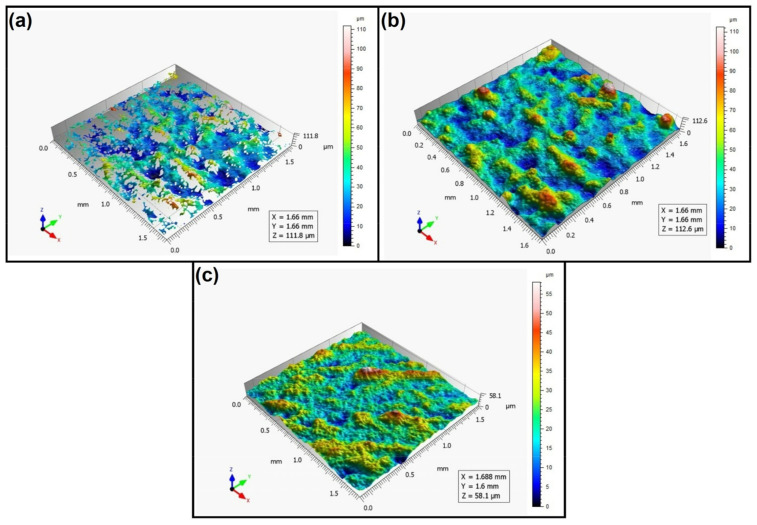
Surface topography of the element 3D printed using SLM technology (Pd = 0°). (**a**) Talysurf CCI—without filling non-measured points, (**b**) Talysurf CCI—after filling non-measured points, (**c**) Talysurf PGI 1230.

**Figure 13:**
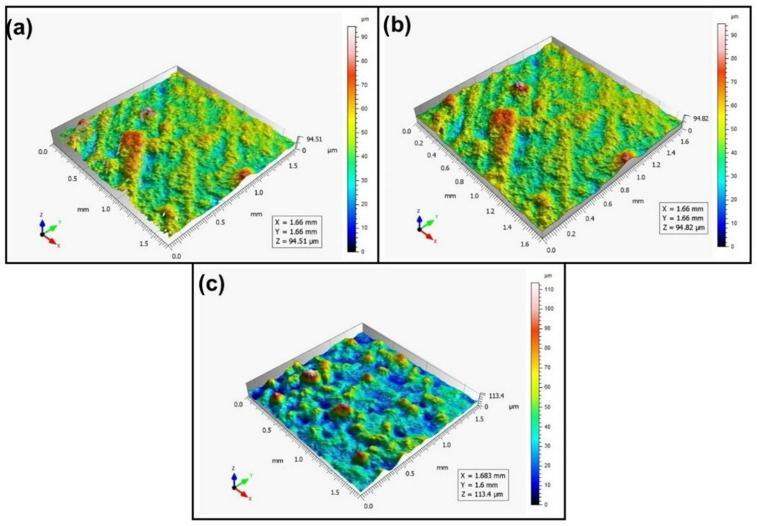
Surface topography of the element 3D printed using SLM technology (Pd = 45°). (**a**) Talysurf CCI—without filling non-measured points, (**b**) Talysurf CCI—after filling non-measured points, (**c**) Talysurf PGI 1230.

**Figure 14:**
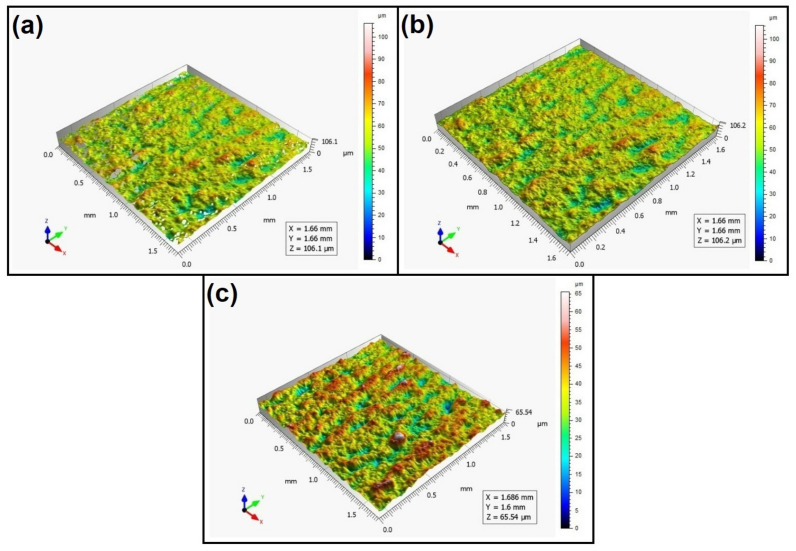
Surface topography of the element 3D printed using SLM technology (Pd = 90°). (**a**) Talysurf CCI—without filling non-measured points, (**b**) Talysurf CCI—after filling non-measured points, (**c**) Talysurf PGI 1230.

To visually assess the surface topography of elements printed with selected additive technologies, views of the surface topography obtained using two measuring systems (i.e., Talysurf CCI and Talysurf PGI 1230 are presented in the below figures, Figure 3, Figure 4, Figure 5, Figure 6, Figure 7, Figure 8, Figure 9, Figure 10, Figure 11, Figure 12, Figure 13 and Figure 14). It should be noted that the Talysurf PGI 1230 measurement system utilizes the contact measurement method and, in this case, 100% of the measured values were assumed.

When analyzing the views of the surface topography obtained using the Talysurf CCI optical profilometer (Figure 3a, Figure 4a, and Figure 5a), one can see that the surfaces were not measured. This was confirmed by the results presented in Table 2. The procedure of filling unmeasured points gives a misleading idea of the measured area (see Figure 3b, Figure 4b, and Figure 5b). However, when analyzing the isometric images of the surface topography obtained by using the Talysurf PGI 1230 contact profilometer, one can note a clear structure distinguished by a large number of individual peaks and valleys. For each analyzed printing direction, the characteristics of the surface irregularities were similar. This is due to the way the SLS technology is implemented by the 3D printer. For each printing direction, a layer of material (powder) is sintered by a laser beam.

When analyzing the views of the surface topography of elements printed using FDM technology, one can periodically see occurring hills, related to the direction of the printing material application. When analyzing Figure 6a, Figure 7a, and Figure 8a, we can see that only the top surface of the peaks was measured. Detailed measurement of the surface of the valleys was difficult. However, the structure obtained for the angle of Pd = 90° (see Figure 8) differed from the surface topography of the elements printed at the angles Pd = 0° and Pd = 45°. In this case, more “flattened” surfaces were obtained. It should be added that the largest number of measured points was obtained for this printing direction.

When analyzing the surface topography view for the samples printed of liquid resin using PJM Technology, one can see a clear directionality of the structure depending on the printing direction. Similarly to FDM technology, periodically occurring peaks can also be seen here (Figure 9, Figure 10 and Figure 11). In addition, in the case of PJM Technology, in the print direction Pd = 90° (Figure 11), for example, local traces of material curing initiated by UV light were visible.

When analyzing the views of the surface topography presented in Figure 12, Figure 13 and Figure 14, one can see that the nature of the surface irregularities of the metal samples printed using SLM technology was similar to the surface of the elements printed using SLS technology. This was due to the similarity of the operation of the printer in both of the analyzed technologies. In SLS technology, the plastic powder is sintered by a laser, while in SLM technology, metal powder is sintered by a laser. However, the surfaces made of metal powders were better mapped than those made of polyamide. This is due to the fact that metal surfaces are more reflective, while the polyamide surface diffuses mode light, which directly affects the number of unmeasured points.

## 4. Conclusions

The intensive development of additive technologies makes them more and more popular, and in the future, they may replace conventional manufacturing technologies. The current state-of-the-art of 3D printers allows for the production of functional machine parts. Therefore, the accuracy of the 3D printing of such elements should be examined in detail. However, in the case of responsible machine parts, the quality of the surface layer should also be tested by measuring and analyzing the surface stereometry parameters.

Due to the different methods of 3D printing as well as the different types of material used to print the elements, sometimes, the assessment of surface topography using non-contact measuring systems is difficult to perform or even impossible. Therefore, the main purpose of the research described in this paper was to analyze the selected measurement factors (i.e., the number of non-measured points to the values of surface topography parameters).

The results of the research presented in this paper prove that interferometric methods may not always be useful for assessing the surface topography of elements printed using some methods of additive technologies. Among all of the analyzed additive technologies, namely, SLS, FDM, PJM, and SLM, only in the case of PJM Technology and the printing angle of Pd = 90° was the surface measured with relatively high accuracy. The number of non-measured points was only 1.5%. For the remaining additive technologies analyzed, the number of non-measured points ranged from 30.1% to 99.6%. The values were significant. The worst results were obtained when using the technology of the selective laser sintering of metal powders, where, depending on the printing direction, the number of non-measured points was in the range of 98.8–99.6%, which excludes the analyzed measurement method for analyzing the surface topography of elements printed using SLS technology.

By analyzing the values of topography parameters before and after applying the procedure of filling non-measured points, it can be concluded that this function reduces the values of the tested surface topography parameters. This reduction depends on the type of additive technology used, the technological parameters, and the number of non-measured points.

Therefore, in the case of a large number of non-measured points, the use of the MountainsMap^®^ software option called “smooth shape calculated from the neighbors—optimize for space points” may provide an unreliable estimate of the surface topography.

When analyzing the values of the surface topography parameters as a whole, it can be concluded that the best results were obtained for PJM Technology and the printing direction of Pd = 90°, where the value of the parameter Sa = 1.07 µm was obtained. Moreover, for the analyzed additive technologies, the lowest values of the topography parameters were obtained for the Pd = 90° printing direction. However, the use of this direction is associated with a longer manufacturing time, which in some cases is unprofitable.

As the aim of further research, the authors will analyze other methods of measuring the surface topography of elements printed using additive technologies. Among other things, atomic force microscopy (AFM) will be used for these types of measurements. In addition, we plan to analyze other factors that may affect the quality of the measured surface mapping (e.g., the type of light used, the method of removing support material from the 3D printed samples, etc.).

## Figures and Tables

**Table 1:** Selected mechanical properties of the materials used to print the samples [18,27,28].

Type of Additive Technology	Material	Tensile Strength, MPa	Young’s Modulus, MPa	Hardness
PJM	FullCure 720	60	2870	83 (Shore scale D)
FDM	ABS P430	37	2320	76 (Shore scale D)
SLS	PA2200	48	1700	75 (Shore scale D)
SLM	316 L grade steel	615	200·10^3^	20 HRC

**Table 2:** Measurement results obtained for the Talysurf CCI optical profilometer.

Technology		SLS			PJM			FDM			SLM		
Printing direction, Pd		0°	45°	90°	0°	45°	90°	0°	45°	90°	0°	45°	90°
Non-measured points,%		99.6	99.6	98.8	9.91	12.2	1.5	88.8	89.8	60.4	72.9	30.1	40.7
Sa, µm	Before filling	22.2	21.46	20.50	3.23	3.96	2.03	9.5	2.91	6.17	10.55	6.08	5.27
	After filling	16.18	17.46	19.03	3.22	4.02	2.04	8.69	3.19	7.46	11.81	6.36	5.30
Sq, µm	Before filling	29.00	26.66	26.72	3.59	4.51	2.47	12.15	3.59	7.87	14.43	8.24	6.67
	After filling	20.93	22.46	23.82	3.6	4.56	2.47	11.06	4.18	9.79	15.54	8.56	6.75
Sz, µm	Before filling	200.7	217.4	181.0	41.88	30.72	46.44	72.36	54.65	76.82	111.8	94.51	106.1
	After filling	198.1	207.2	178.7	41.88	30.77	46.41	69.01	54.99	77.01	112.6	94.82	106.2
Ssk	Before filling	−1.01	−0.45	1.07	0.19	0.65	0.23	−0.19	−0.19	−0.38	1.42	0.94	−0.02
	After filling	−0.72	−0.44	0.52	0.25	0.54	0.21	0.15	−0.77	−0.87	1.08	0.89	−0.06
Sku	Before filling	4.67	3.58	3.58	1.73	2.01	2.85	2.63	4.04	4.00	6.87	5.37	3.37
	After filling	4.79	3.96	2.9	2.06	1.92	2.81	2.76	5.47	4.05	4.85	5.15	3.30

**Table 3:** Measurement results obtained for the Talysurf PGI optical profilometer 1230.

Technology Name	SLS			PJM			FDM			SLM		
Printing direction	0°	45°	90°	0°	45°	90°	0°	45°	90°	0°	45°	90°
Sa, µm	15.75	19.36	15.73	1.89	2.65	1.07	21.17	30.45	8.68	5.68	11.1	5.59
Sq, µm	19.93	23.92	19.92	2.52	3.08	1.32	25.2	35.65	10.73	7.23	14.78	7.13
Sz, µm	167.2	190	153.1	18.57	19.05	9.18	116.9	148.1	63.20	58.10	113.4	65.54
Ssk	−0.24	0.09	0.03	0.21	0.14	0.00	−0.41	−0.69	−0.55	0.64	1.16	−0.05
Sku	3.29	2.74	2.91	3.68	1.98	2.68	2.25	2.27	3.07	3.94	5.56	3.39

## Data Availability

Not applicable.

## References

[1] Wieczorowski M., Oliwa R., Woźniak J., Sobolewski B., Paszkiewicz A., Przeszłowski Ł., Oleksy M., Budzik G. (2022). The Place of 3D Printing in the Manufacturing and Operational Process Based on the Industry 4.0 Structure. Teh. Glas..

[2] Kozior T., Döpke C., Grimmelsmann N., Juhász Junger I., Ehrmann A. (2018). Influence of Fabric Pretreatment on Adhesion of Three-Dimensional Printed Material on Textile Substrates. Adv. Mech. Eng..

[3] Yan Q., Dong H., Su J., Han J., Song B., Wei Q., Shi Y. (2018). Additive Manufacturing—Review A Review of 3D Printing Technology for Medical Applications. Engineering.

[4] Kozior T., Bochnia J., Gogolewski D., Zmarzły P., Rudnik M., Szot W., Szczygieł P., Musiałek M. (2022). Analysis of Metrological Quality and Mechanical Properties of Models Manufactured with Photo-Curing PolyJet Matrix Technology for Medical Applications. Polymers.

[5] Adamczak S., Zmarzly P., Kozior T., Gogolewski D. Analysis of the dimensional accuracy of casting models manufactured by fused deposition modeling technology. Proceedings of the 23rd International Conference on Engineering Mechanics 2017.

[6] Blasiak S., Laski P.A., Takosoglu J.E. (2021). Rapid Prototyping of Pneumatic Directional Control Valves. Polymers.

[7] Borawski A. (2021). Impact of Operating Time on Selected Tribological Properties of the Friction Material in the Brake Pads of Passenger Cars. Materials.

[8] Bochnia J., Blasiak M., Kozior T. (2020). Tensile Strength Analysis of Thin-Walled Polymer Glass Fiber Reinforced Samples Manufactured by 3d Printing Technology. Polymers.

[9] Kozior T. (2020). Rheological Properties of Polyamide Pa 2200 in Sls Technology. Teh. Vjesn..

[10] Hatz C.R., Msallem B., Aghlmandi S., Brantner P., Can F.M.T. (2019). Can an Entry-Level 3D Printer Create High-Quality Anatomical Models ? Accuracy Assessment of Mandibular Models Printed by a Desktop 3D Printer and a Professional Device. Int. J. Oral Maxillofac. Surg..

[11] Łukaszewicz A., Miatliuk K. (2009). Reverse Engineering Approach for Object with Free-Form Surfaces Using Standard Surface-Solid Parametric CAD System. Solid State Phenom..

[12] Mathia T.G., Pawlus P., Wieczorowski M. (2011). Recent Trends in Surface Metrology. Wear.

[13] Galy C., Le Guen E., Lacoste E., Arvieu C. (2018). Main Defects Observed in Aluminum Alloy Parts Produced by SLM: From Causes to Consequences. Addit. Manuf..

[14] Skrzyniarz M., Nowakowski L., Blasiak S. (2022). Geometry, Structure and Surface Quality of a Maraging Steel Milling Cutter Printed by Direct Metal Laser Melting. Materials.

[15] Ma Q.P., Mesicek J., Fojtik F., Hajnys J., Krpec P., Pagac M., Petru J. (2022). Residual Stress Build-Up in Aluminum Parts Fabricated with SLM Technology Using the Bridge Curvature Method. Materials.

[16] Podulka P. (2022). Advances in Measurement and Data Analysis of Surfaces with Functionalized Coatings. Coatings.

[17] Feng X., Senin N., Su R., Ramasamy S., Leach R. (2019). Optical Measurement of Surface Topographies with Transparent Coatings. Opt. Lasers Eng..

[18] Adamczak S., Zmarzly P., Kozior T., Gogolewski D. Assessment of roundness and waviness deviations of elements produced by selective laser sintering technology. Proceedings of the 23rd International Conference on Engineering Mechanics 2017.

[19] Zmarzły P., Adamczak S. The Evaluation of a Contact Profilometer Measuring Tip Movement on the Surface Texture of the Sample. Proceedings of the International Symposium for Production Research.

[20] Podulka P. (2022). Thresholding Methods for Reduction in Data Processing Errors in the Laser-Textured Surface Topography Measurements. Materials.

[21] Leach R.K. (2011). Optical Measurement of Surface Topography.

[22] Pawlus P., Reizer R., Wieczorowski M. (2017). Problem of Non-Measured Points in Surface Texture Measurements. Metrol. Meas. Syst..

[23] Adamczak S., Kundera C., Swiderski J. (2017). Assessment of the State of the Geometrical Surface Texture of Seal Rings by Various Measuring Methods. IOP Conf. Ser. Mater. Sci. Eng..

[24] Merola M., Ruggiero A., Salvatore J., Mattia D., Affatato S. (2016). On the Tribological Behavior of Retrieved Hip Femoral Heads Affected by Metallic Debris. A Comparative Investigation by Stylus and Optical Profilometer for a New Roughness Measurement Protocol. Measurement.

[25] Koizumi H., Maki A., Yamamoto T. Optical Topography: Practical Problems and Novel Applications. Proceedings of the Optics InfoBase Conference Papers.

[26] Grzejda R., Parus A., Kwiatkowski K. (2021). Experimental Studies of an Asymmetric Multi-Bolted Connection under Monotonic Loads. Materials.

[27] Bochnia J. (2018). Evaluation of Relaxation Properties of Digital Materials Obtained by Means of PolyJet Matrix Technology. Bull. Polish Acad. Sci. Tech. Sci..

[28] Hardes C., Pöhl F., Röttger A., Thiele M., Theisen W., Esen C. (2019). Cavitation Erosion Resistance of 316L Austenitic Steel Processed by Selective Laser Melting (SLM). Addit. Manuf..

